# Overburden, Stigma, and Perceived Agency: Teachers as HIV Prevention Educators in Urban Zambia

**DOI:** 10.3934/publichealth.2016.2.265

**Published:** 2016-05-05

**Authors:** Margaret Henning, Sunil K. Khanna

**Affiliations:** 1. Health Science, Keene State College, Keene NH; 2. School of Biological and Population Health Sciences, College of Public Health and Human Sciences, Oregon State University.

**Keywords:** HIV/AIDS, Educators, Zambia, Prevention, School-based programs

## Abstract

Sub-Saharan Africa is home to more than 70% of the global HIV-positive population. In Zambia, as well as in other parts of Africa, deaths from AIDS and associated infections have created a generation of households headed by children, a situation that negatively affects the chances for economic and health improvements in the region. In contemplating possible public health interventions around HIV prevention, we found that a growing body of research advocates for school-based HIV programs as an effective strategy to stop the spread of the disease. This work is critical because it explores schoolteachers' perspectives on their potential roles as HIV prevention educators. Semi-structured interviews (n = 12) were conducted among schoolteachers in the Lusaka province of Zambia to collect qualitative data. Analysis of qualitative data revealed three broad and interconnected themes related to the roles and concerns of the participating teachers: 1) the role of overburden; 2) fear of stigma; and 3) perceived lack of agency. These themes are further discussed in the context of the results that focused on the teachers and the adoption of HIV education. Little is known about teachers' perceptions of themselves as HIV educators. Our study suggests that understanding teachers' perceptions and the contextual factors is crucial to the adoption of school-based HIV programs.

## 1. Introduction

An estimated 30 million people are living with HIV worldwide; 22.5 million live in sub-Saharan Africa[1]. As of 2009, the estimated HIV prevalence rate in Zambia is 13.5%. Mortality from AIDS and associated infections has created a generation of orphans and vulnerable children (OVC) — school census conducted by the Ministry of Education (MoE) in 2007 estimated the total number of orphans at 1.3 million. According to the 2007 Zambian Demographic Health Survey, 14.9% of all orphan children are vulnerable to HIV[2]. This growing number negatively affects and further exasperates the chances for economic and health improvements in the region. A growing body of research suggests that school-based HIV programs are a crucial step [3],[4] to curb the rising tide of HIV infection. Education levels are a strong predictor of HIV knowledge, safer sexual behavior, and reduced HIV infection rates[5]. de Walque (2009) asserts that schooling is one of the most consistent predictors of behavior and knowledge[6].

Schools provide a critical opportunity to address one of the major HIV/AIDS health challenges that faces Zambia[7]. However, research on HIV/AIDS education in schools generally focuses on assessing change in school children — the core target group [8],[9]. Little attention has been has been paid to the role of schoolteachers' as HIV educators. Because teachers have a generally higher social status in a community and a high degree of contact with students and parents, they can be effective stakeholders in the fight against HIV/AIDS. Furthermore, research suggests that HIV interventions HIV should target family and societal domains; as such schools need to be studied as a social domain[10].

The purpose of this study was to elicit schoolteachers' perspectives on their potential roles as HIV prevention educators. By eliciting HIV education narratives from teachers engaged in instruction across a spectrum of school types (public, private and community) in Zambia, we sought to identify common themes in the lived experiences of participants. By understanding the key barriers to, and benefits of, school-based HIV prevention education we elucidate culturally appropriate steps to improve the efficacy of this approach to reducing HIV infection. Here we discuss three key barriers identified in teachers' narratives and from data collected through surveys. These are i) overburden, ii) fear of stigma, and iii) a perceived lack of agency as central themes.

### 1.1. Education System in Zambia

Our research is situated within the specific context of gender- and age-based factors that influence the spread of HIV in Zambia. Zambia's education system includes basic education (grades 1 through 9), high school (grades 10 through 12), and tertiary education (university or college). Students are enrolled in three tiers of schools within the educational structure. The first tier includes private schools, which are often operated with religious affiliations. These schools require fees and payments for books and uniforms. The second tier includes basic public or government schools (GRZ). These schools are part of the government's education policy to promote education.

The last tier includes community schools, a component of an education system that evolved when communities decided to address their children's lack of education, due in part to economic restraints. Communities manage these schools locally although they are still under the auspices of the Ministry of Education (MOE). Teachers in community schools are community members without formal training and are paid in-kind donations (chickens, food, or other goods). A typical community school building may have two or three small classrooms under shelter, in addition to outside classrooms. Recent estimates suggest that of the 3.2 million children enrolled in basic-level, primary education in Zambia, 77% attend government schools, and 16% and 7% attend community and private schools, respectively (IOB Impact Evaluation, 2008).

### 1.2. Age and Gender

The HIV prevalence in Zambia was estimated to be at 13.5% as of 2009 and women are more likely to be living with HIV (60% women and 40% men, respectively). In Zambia, the decline in the prevalence rate for 15 to 19-year-old women in Lusaka was more substantial for those with secondary and higher levels of education than for those without a primary school education[11]. Although it is important to focus on people in early adulthood, it is regrettable that similar prevention attention is not paid to younger children in school who have not been infected. Those often described as the “window of hope”[12],[13].

## 2. Methods

### 2.1. Ethical Approval

The Oregon State University Institutional Review Board and the University of Zambia Ethical Review Board approved this work. First, information about the schools was obtained from an Education Management Information Systems (EMIS) Ministry of Education special data request (2006).

### 2.2. Study Sample

In-depth interviews were conduced with eleven school teachers. All teachers were at least 18 years of age. The study population was not restricted by gender. School deputies or administrators were contacted for their permission before we made any contact with schoolteachers. Interviews with teachers were from each school type (private/church, community, and government).

The primary author of this paper personally conducted all of the interviews. At the beginning of the interview, the researcher read through the consent document with each participant and received verbal consent as required by the by the Oregon State University Institutional Review Board and the Zambian Ministry of Education.

The study adopted a qualitative approach to gain an in-depth understanding of the experiences of school teachers and their role as HIV educators[14],[15].

Snowball sampling was used for this work to build an exhaustive sampling frame from which a random sample can be drawn. This method can produce useful non-probability samples of selected members from a specific community or interest group[16]. All interviews were conducted in English. Initial research questions were open-ended to explore key issues in depth. Follow-up questions afforded limited choices without compromising on the possibility of further discovery. Open-ended responses extended the balance and expertise from the researcher to the respondents, in this case teachers, thus encouraging them to identify and articulate their priorities[17]. Generally speaking, in qualitative methods one continues to interview individuals until a point of―concept saturation is achieved. When themes started repeating, the researcher drew the process to a close[18]. The average interview time was between an hour to an-hour-and-half.

### 2.3. Analysis

Qualitative data were analyzed in three main phases (1) open coding, (2) searching for themes, (3) drawing conclusions. Interviews were transcribed to best represent the dynamic nature of the conversation[19]. Data analysis of the individual interviews began with “open coding”; data were explored without assumptions and to allow for surprises[20]. This step helped to conceptualize and categorize the data[20]. Open coding allows for more powerful categories and included rich descriptions from the participants. As a result, multiple themes emerged and were analyzed. Quotes from several informants were used to triangulate findings, discuss results, and draw conclusions.

## 3. Results and Discussion—Emergent Themes

The average age of the sample teachers was 35 years with an average of 12 years of formal education. Community schoolteachers tended to be younger and had fewer years of education (10.5 years on average). Since community schools teachers are usually paid in-kind and are relatively informant, school teachers receive less formal training than those in government or private schools.

Finally, the teachers' individual responses were grouped into common categories based on the number of times and the depth of each category — i) overburden, ii) fear of stigma, and iii) perceived lack of agency. Themes were not necessarily dependent on quantifiable measures.

**Table 1.** Major Themes: School Teachers' narritives in their role as educators and promoters of HIV/AIDS prevention education.

Theme	Description
Overburden	Expression of severity and intensity of the HIV epidemic, descriptions of the experiences, feelings, and perceptions that related to their role in HIV/AIDS prevention and support to students and communities.
Stigma	Descrptions of self-experiences of stigmatization or with stigmatization of student.
Lack of Agency	Identified beliefs associated with HIV/AIDS, outcome of HIV/AIDS education, and social responsibility; school teachers' perception of agency within the community in their role as HIV/AIDS educator.
Self-efficacy (specific to teaching HIV prevention)	Descriptions of concerns related to lack of skills to promote HIV/AIDS prevention.
Structural Factors	Idenified policy and envrioment issues, such as lack of support services, resources, etc.

### 3.1. Overburden

Assuming that teachers can play the role of caregivers in schools, overburden appears that a common barrier to teaching HIV prevention. According to Van Dyk's (2007) study on occupational stress experienced by HIV/AIDS caregivers in South Africa, teachers cited substantially more symptoms of occupational stress than counselors and health care workers[21].

In our sample, school teachers expressed a feeling of being overwhelmed by the needs of students, and expressed concern as to how they will be perceived by parents of students. This concern primarily focused on parents' negative perception of school teachers discussing HIV/AIDS and sexual health related topics with students. In particular, teachers were concerned about how their role as teachers would be seen as inappropriate considering religious and cultural norms in the community. As one government schoolteacher said,

“Parents do support our HIV education, but still some parents will not talk to their children about such things, even if they are living with HIV in the home.” *Male, age 31*

There is also concern on the part of teachers for how parents or caregivers might react to teachers addressing the topic of HIV/AIDS prevention.

“You know as a teacher, I worry. I know students need to know such information [HIV prevention]. But what if a parents get mad for what we are teaching…I already teach many topics now I have to find a way to teach this. Yes, it is important but we are not talking to the parents about the ways we should be teaching this.” *Felmale, age unknown*

Feeling overburden with teaching and the added responsibilities of serving as HIV/AIDS educators has been reported as a common barrier in sub-Saharan Africa with health workers delivering HIV prevention[22]. Our findingds support this claim—school teachers reported barriers such as stress due to less time, poor knowledge and training for their role as HIV/AIDS educators, and overburdened workload. School teachers also identified fear exposure to HIV and inability to address confidentiality issues regarding their own HIV status or that of their students as other barriers preventing them to be effective HIV/AIDS educators.

### 3.2. Stigma

According to Goffman (1963) stigma is a process of social construction. This relationship further connects stigma to the underlying theory (social construction) used to understand teachers' perceptions [23]. Kelly and Bain (2003) suggest that Stigma is an obstacle to both prevention and care[24]. Stigma creates a context where people are divided by distinguishing and labeling differences, associating negative attributes and those discredited are regarded as the “others.” The social domain of teachers and their expressions about, or descriptions of situations suggest that HIV stigma does exist and thus affects their role as an educator.

Schools teachers admitted that stigma associated with HIV/AIDS may lead to social isolation or rejection of an afflicted individual. However, school teachers suggested that this cultural dynamic is gradually shifting. Community members are increasing becoming more accepting and less discriminatory toward individuals living with HIV/AIDS.

As a community schoolteacher suggested,

“We all know that someone's sister or brother or friend has been diagnosed as HIV positive. We cannot discriminate against those who we know or care for. We have to be more accepting of them.” *Female, age 28*

Although school teachers expressed that the social climate surrounding HIV stigma is gradually changing, several teachers shared their experiences of how stigma has shaped their own lives. According to a government schoolteacher,

“You know I am HIV positive. It was so hard when I learned about this. I have only told my friends—slowly, they talk with me as they too are struggling with HIV either themselves or in their family. We need to have a support group. I remember the day I found out; when I was pregnant they tested me. I did not tell him (husband) for months, I was scared. I remember the day I told him. He left the house and did not come back for a long time. When he did return, we decided I needed to leave. So I left to live with my mother and daughter.” *Female, age 26*

In the words of another school teacher,

“We teach about stigma this is good and we need change, really talking about …. You know it is different than acting on really addressing stigma. Schools that have anti-discrimination policies and support for a child or teacher, by providing support groups these schools are addressing the challenge.” *Female, age 33*

At the larger community and cultural levels, it is important to understand the role of stigma that teachers may face to fully comprehend their contributions to promote the well-being of children[25]. It is critical to recognize that teachers are embedded within a complex social system in which their roles as educators are often perceived by community members in moral terms. Teachers want to teach and provide HIV prevention in a way that is acceptable within the local social context. Our study underscores the importance of HIV anti-discrimination policy and the need to have resources and support for teachers to address HIV prevention education in a way that will meet their individual needs, but also the needs and well-being of their students.

### 3.3. Lack of Agency

Our in-depth interviews suggest that school teachers who had prior experience of teaching topics related to HIV/AIDS prevention tended to express positive attitudes toward teaching HIV/AIDS. On the contrary, those teachers who had less or no experience expressed “feeling embarrassed” while discussing HIV/AIDS related issues with students. These findings reinforce the pre-existing knowledge on this topic. Several studies suggest that school teachers often do not address some of the major HIV/AIDS prevention issues in part because of fear of community disapproval and controversy, as well as lack of supportive guidance[26].

In alignment with others studies on this topic[27], our study suggests that many teachers may have formal or informal knowledge about HIV/AIDS prevention, but may not specifically know how or what topics to teach to students. They may lack sufficient knowledge of effective pedagogy to teach HIV/AIDS prevention. Importantly, school teachers also expressed a strong desire to be accepted by the local community as HIV/AIDS educators, thus reinforcing the earlier observation that for the school teachers to be effective HIV/AIDS educators, community acceptance plays an important role.

As one teacher expressed,

“Teaching this (HIV/AIDs prevention) is important, we have this in our mission statement, but if I know parents will be angry, I want to keep my job you see, what is the motivation for me? I care, but I also need to feel supported by the community, by the parents. They might be upset for what I tell their children.” *Male, age 27*

Clearly, this study suggests that teachers work in a complex social and professional environment that includes the local community and their commitment to education and health of their studetns. The teachers are well positioned to address sensitive issues related to HIV/AIDS prevention. However, they need support in terms of formal training on HIV/AIDS prevention, effective pedagogy and teaching skills, and acceptance in the local community for their role as educators, in general, and HIV/AIDS prevention educators, in particular.

## 4. Discussion and Conclusion

In this study we have identified three emergent and overlapping themes that contextualize the everyday “lived and shared” experience of schoolteachers. We argue that understanding schoolteachers' experiences of overburden, stigma, and lack of agency can help us plan effective and sustainable HIV intervention programs.

Our study suggests that schoolteachers are well-positioned and are willing to support HIV education in schools and that school, in essence, can be an ideal environment to systematically deliver information. However, for effective HIV program planning and implementation it is important to consider the workload and experiences of schoolteachers. In addition, schoolteachers' perceived concern for stigma is intricately linked with their role as educators and how they believe the community will view them as key players in efforts to prevent the spread of HIV. Western approaches often focus on individuality; this position is seemingly not as appropriate for an educator in Zambia, where his or her role as HIV educator is contingent on community perceptions and support. Thus, an understanding of a society's cultural particulars, especially as they relate to socially permissible discourse on sex as well as the social status and roles of educators, can be helpful in planning effective HIV intervention programs. Finally, understanding the factors that influence schoolteachers' traditional responsibilities in addition to the teaching of HIV prevention can be useful to develop programs and HIV-prevention educator trainings.

Specifically relative to Zambia, schoolteachers indicated a willingness to teach HIV prevention, however willingness needs to be combined with high levels of HIV/AIDS knowledge — as such, increased training that goes beyond basic prevention education. For example, teacher-training programs should focus on counseling skills, antiretroviral therapy (ARV), and first aid (schools may sometimes inadvertently become the location where students are exposed to bodily fluids, e.g., blood). Consideration and preference also need to be given to a teacher's existing work allocation, the perceived stigma, and the level of community support when coordinating and prioritizing the role of schoolteachers in providing essential HIV education. We strongly recommend the need to account for the contextual environment that each teacher works in [28]. These warrant attention, as schools increasingly become a point of health care delivery.

There are several limitations to this study. First, only the schoolteachers' perspectives are taken into consideration, and not those of the students. This work included an overrepresentation of men. We suggest that future studies should examine perceptions of the community regarding teachers as resources for HIV prevention. This article is based on in depth data collected during 12 interviews with schoolteachers. Although we were able to achieve concept saturation in our qualitative study in terms of key themes identified in this article, we do not claim that our results can be generalizable to the entire population of schoolteachers in the country. In other words, the results presented here simply shed light on the shared experiences of those schoolteachers who participated in our study. Finally, this work recognizes that actions related to HIV/AIDS prevention are decided locally, are culturally appropriate, and that the community chooses their own priorities.
